# Resistance to Sulfuric Acid Corrosion of Geopolymer Concrete Based on Different Binding Materials and Alkali Concentrations

**DOI:** 10.3390/ma14237109

**Published:** 2021-11-23

**Authors:** Wei Yang, Pinghua Zhu, Hui Liu, Xinjie Wang, Wei Ge, Minqi Hua

**Affiliations:** 1 Department of Civil Engineering, Changzhou University, Changzhou 213164, China; cczuyw@163.com (W.Y.); zph@cczu.edu.cn (P.Z.); wangxinjie@cczu.edu.cn (X.W.); 2 Department of Materials Science and Engineering, Anhui University of Science and Technology, Huainan 232001, China; 3 School of Civil Engineering and Architecture, Wuhan University of Technology, Wuhan 430070, China; alcestle@gmail.com

**Keywords:** geopolymer concrete, fly ash, metakaolin, alkali concentration, sulfuric acid corrosion

## Abstract

Geopolymer binder is expected to be an optimum alternative to Portland cement due to its excellent engineering properties of high strength, acid corrosion resistance, low permeability, good chemical resistance, and excellent fire resistance. To study the sulfuric acid corrosion resistance of geopolymer concrete (GPC) with different binding materials and concentrations of sodium hydroxide solution (NaOH), metakaolin, high-calcium fly ash, and low-calcium fly ash were chosen as binding materials of GPC for the geopolymerization process. A mixture of sodium silicate solution (Na_2_SiO_3_) and NaOH solution with different concentrations (8 M and 12 M) was selected as the alkaline activator with a ratio (Na_2_SiO_3_/NaOH) of 1.5. GPC specimens were immersed in the sulfuric acid solution with the pH value of 1 for 6 days and then naturally dried for 1 day until 98 days. The macroscopic properties of GPC were characterized by visual appearance, compressive strength, mass loss, and neutralization depth. The materials were characterized by SEM, XRD, and FTIR. The results indicated that at the immersion time of 28 d, the compressive strength of two types of fly ash-based GPC increased to some extent due to the presence of gypsum, but this phenomenon was not observed in metakaolin-based GPC. After 98 d of immersion, the residual strength of fly ash based GPC was still higher, which reached more than 25 MPa, while the metakaolin-based GPC failed. Furthermore, due to the rigid 3D networks of aluminosilicate in fly ash-based GPC, the mass of all GPC decreased slightly during the immersion period, and then tended to be stable in the later period. On the contrary, in metakaolin-based GPC, the incomplete geopolymerization led to the compressive strength being too low to meet the application of practical engineering. In addition, the compressive strength of GPC activated by 12 M NaOH was higher than the GPC activated by 8 M NaOH, which is owing to the formation of gel depended on the concentration of alkali OH ion, low NaOH concentration weakened chemical reaction, and reduced compressive strength. Additionally, according to the testing results of neutralization depth, the neutralization depth of high-calcium fly ash-based GPC activated by 12 M NaOH suffered acid attack for 98 d was only 6.9 mm, which is the minimum value. Therefore, the best performance was observed in GPC prepared with high-calcium fly ash and 12 M NaOH solution, which is attributed to gypsum crystals that block the pores of the specimen and improve the microstructure of GPC, inhibiting further corrosion of sulfuric acid.

## 1. Introduction

Concrete durability refers to the resistance of concrete against the action of aggressive environmental media which threaten the normal service of concrete components [1]. The actions of these aggressive environmental media are usually summarized as acid attack [2], sulphate attack [3], and chloride-induced corrosion [4]. In the different forms of concrete durability failure, acid attack is the main factor affecting the service life of concrete used in sewage collection systems. The lifespan of concrete used for these wastewater networks significantly reduces to around 30–50 years from the design life of 100 years, which is attributed to acid attack caused by bacterial activity [5,6,7]. In addition, the acid attack of concrete has contributed to huge economic losses worldwide [8]. Thus, it is too impatient to wait to improve the acid resistance durability of concrete used for wastewater networks.

Geopolymer, as some novel binder materials, due to its excellent engineering properties of high strength, high temperature resistance, weather resistance, low permeability, and acid corrosion resistance [9,10,11], has been studied extensively. Geopolymer concrete (GPC) is composed of aggregates and alkali-activated aluminosilicate materials, such as aluminosilicate materials including fly ash (FA), metakaolin (MK), and ground granulated blast furnace slag (GGBS), etc. [12,13,14]. Under the action of an alkali activator, GPC undergoes geopolymerization and generates an amorphous three-dimensional network structure of silicon-oxygen tetrahedron and aluminum-oxygen tetrahedron connected through bridge oxygen [15,16,17], which endows the GPC with better acid corrosion resistance when compared with the OPC concrete [18]. It is reported that the acid corrosion resistance of OPC mainly depends on the hydration product and the quality of the protective layer [19,20]. However, for GPC, it is the depolymerization of aluminosilicate polymers and the liberation of silicic acid which affect its acid corrosion resistance [20,21]. Accordingly, the acid corrosion resistance of GPC is superior to OPC concrete for its more stable cross-linked aluminosilicate polymer structure in GPC.

In studies so far, there are a large number of scholars that have studied the acid resistance of GPC. They found that the acid degradation of the calcium-free geopolymer (metakaolin) begins with an ion-exchange between framework cations (i.e., sodium) and protons from the acid solution. The protons induce an electrophilic attack, which results in the ejection of aluminum (i.e., dealumination) from the Si-O-Al bonds of the binder [22]. Timothy et al. [23] studied the acid degradation mechanism of low-calcium fly ash binders and demonstrated its similar destruction process with a calcium-free geopolymer. The only difference is that the diffusing SO_4_^2−^ anions meet with counter diffusing calcium ions, causing the deposition of gypsum crystals inside the penetrated layer. Nuaklong et al. [24] used metakaolin as a partial substitution for high-calcium fly ash in geopolymer binders and concluded that the mixed binders exhibited higher resistance to acid attacks than the single binder due to the decline of calcium content of mixtures. However, Mehta et al. [25] investigated the sulfuric acid resistance of high-calcium fly ash-based geopolymer concrete blended with an additional calcium source (OPC). They found that the inclusion of OPC improved the compressive strength of fly ash-based geopolymer concrete specimens significantly and 10% OPC addition exhibited better acid resistance than 0%. Geopolymer mixtures prepared with different binding materials show different resistance to sulfuric acid, especially for calcium-free or calcium-based binding materials. Thus, it is significant to investigate the sulfuric-acid resistance of geopolymers respectively prepared from different binding materials: metakaolin and low-calcium and high-calcium fly ash. Besides, most scholars only take a single concentration of alkali activator into consideration [26,27], ignoring the influence of alkali activator concentration on the acid resistance of geopolymers. Different concentrations of alkali activator should be considered to prepare the geopolymer mixtures.

The work reported herein aimed to clarify the effects of different binding materials and concentrations of alkali activators of GPC on the sulfuric acid corrosion resistance. Additionally, the acid corrosion resistance test in this work was carried out in samples immersed in an acid solution of pH = 1. Wetting-drying cycle experiment was used for the test of acid corrosion resistance. The properties were characterized by visual appearance, compressive strength, mass loss, and neutralization depth. SEM, XRD, and FTIR were selected to analyze the mechanism of acid corrosion resistance for GPC.

## 2. Experimental Details

### 2.1. Raw Materials

Three types of binding materials, including as low-calcium fly ash, high-calcium fly ash, and metakaolin, were adopted to prepare the GPC. The low-calcium fly ash was Class-F, whose content of CaO was less than 10%. The high calcium fly ash was Class-C, and its content of CaO was more than 10%. Metakaolin was purchased from Gongyi, Henan Province. The chemical compositions of the low-calcium fly ash, high-calcium fly ash, and metakaolin were measured by X-ray fluorescence (XRF), as shown in Table 1. The coarse aggregate was natural limestone with the size of 4.75–9.5 mm. Washed river sand was chosen as fine aggregate. The reagents of sodium hydroxide (NaOH ≥ 96.0%, AR), sulfuric acid (H_2_SO_4_ ≥ 95.0%, AR), and phenolphthalein (1%) were purchased from Sinopharm Chemical Reagent Co., Ltd., Shanghai, China. Sodium silicate liquid (Na_2_SiO_3_ with 29.9 wt% SiO_2_, 13.75 wt% Na_2_O, and 56.35 wt% H_2_O) and the distilled water were used in the experiments.

### 2.2. Mix Proportions and Preparation of Specimens

The GPC were prepared with binding materials and an alkaline solution which was a combination of Na_2_SiO_3_ and different concentrations of NaOH (8 M and 12 M) pre-mixed with a ratio (Na_2_SiO_3_/NaOH) of 1.5. The mix proportions for all GPC mixtures are listed in Table 2.

**Figure 1:**
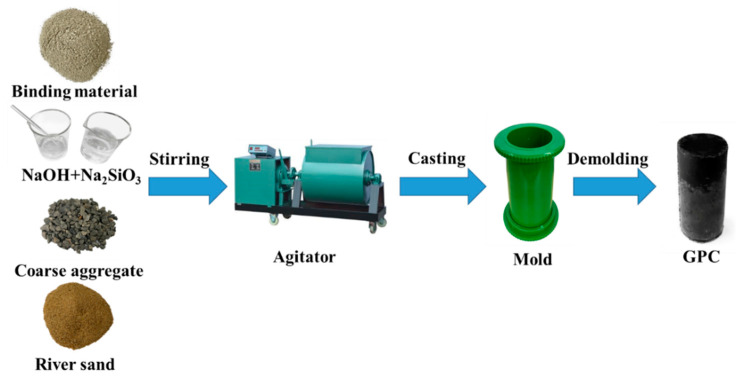
Preparation diagram of GPC.

The preparation process of GPC is shown in Figure 1. The cylindrical specimens with Φ50 × 100 mm in dimension were prepared for tests. It should be noted that the GPC specimens needed to be filled in three layers. After filling, the specimens were vibrated on the vibrating table for 30 s to remove the bubbles inside. Then, they were put into hermetic bags and cured at 60 °C for 48 h. Subsequently, the GPC specimens cooled for 1 h were demolded and preserved in a standard curing room (20 ± 2 °C, RH ≥ 95%) until the age of 7 d. According to the types of binding materials and concentrations of NaOH, the GPC specimens prepared with low-calcium and high-calcium fly ash and metakaolin were marked as F-8, F-12, C-8, C-12, MK-8, and MK-12 respectively.

### 2.3. Measurements

#### 2.3.1. Sulfuric Acid Corrosion Resistance Test

Before the sulfuric acid corrosion resistance test, all the GPC specimens were polished manually on surface using sandpaper to clean up the grease. Then, GPC were coated with vinyl ester resin on the top and bottom face to ensure that only their side faces were exposed to the acid solution. To simulate the actual working environment, all the specimens were immersed in the sulfuric acid solution with pH = 1 for 6 days, and then taken out to dry for 1 day. The whole cycle lasted for 98 days. In addition, it is important to note that the sulfuric acid solution was renewed every week to maintain a relatively stable pH for the solution.

After all types of specimens soaked in the acid solution for 7, 14, 28, 63, and 98 days, the neutralization depth of specimens was tested by spraying 1% phenolphthalein indicator on the section of specimens. The distance between the edge of specimen and discoloration boundary was measured by a vernier caliper. Eight points on each section were selected for measurement, and the arithmetic mean value was taken as the neutralization depth.

The mass loss of GPC specimens in different soaking periods was calculated by the formula:

(1) $$W=\frac{m_{0}-m_{1}}{m_{0}}\times 100\%$$

where, W represents the mass loss rate; m_0_, m_1_ represents the mass of specimens before and after the immersion of sulfuric acid respectively, in g. The time points of measurement were 0, 7, 14, 28, 63, and 98 days.

The compressive strengths of specimens were tested by electro-hydraulic servo universal testing machine (YNS 300) at 0, 7, 14, 28, 63, and 98 days to acid exposure in accordance with ASTM C39/C39M [28]. The value of compressive strength was the average of three specimens.

#### 2.3.2. Microscopic Analysis

For further analysis, all samples were taken from the GPC before and after sulfuric acid exposure.

The morphologies of GPC before and after sulfuric acid exposure were observed using Scanning electron microscopy with energy dispersive X-ray (SEM/EDX, SUPRA55, Zeiss, Oberkochen, Germany) at the accelerating voltage of 15 kV.

An X-ray diffractometer (XRD, D/MAX2500, Rigaku, Tokyo, Japan) was used to analyze the component and crystalline phase variations of GPC. The parameters were set as a voltage of 40 kV, a current of 30 mA, and Cu Kα radiation (k = 0.15418 nm).

Fourier transform infrared spectroscopy (FT-IR) was adopted to characterize the phase compositions of GPC, which was performed on a Thermo Fisher Scientific Nicolet IS50 FT-IR analyzer by using KBr pellet techniques. The resolution and scanning times were 2.0 cm^−1^ and 16 cm^−1^, respectively.

## 3. Results and Discussion

### 3.1. Macroscopic Properties

#### 3.1.1. Visual Appearance

**Figure 2:**
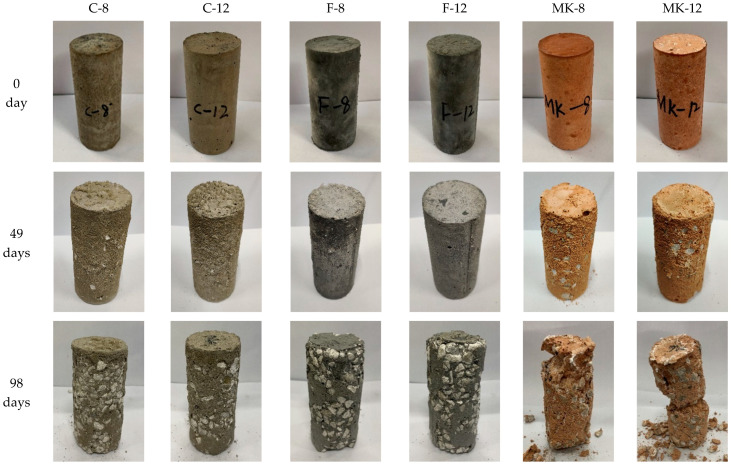
GPC after 0 day, 49 days, and 98 days of immersion in sulfuric acid solution.

Figure 2 shows the visual appearance variation of GPC specimens when exposed in sulfuric acid solution for 0 day, 49 days, and 98 days. The surfaces of all GPC specimens were smooth and flat before the specimens were immersed in the acid solutions. With the increase of exposure time, the damage of specimens became increasingly serious. After 49 days of immersion, the specimens of F-8 and F-12 were almost intact, and minor damage appeared in specimens C-8 and C-12. Macroscopic observations of Mk-8 and MK-12 showed that their structures were visibly loose, and some cracks appeared on the surface and some aggregates were bared, as shown in Figure 2. When the exposure time was up to 98 days, all specimens suffered from varying degrees of damage. The surface of specimens marked with C-8, F-8, and F-12 were rougher due to the spalling of mortar matrix and aggregates, while the MK-8 and MK-12 specimens failed after 98 days of acid corrosion. The observed structure of sample C-12 was intact and dense, in which only a little mortar split away from the surface of the specimen. As it is shown in Figure 2, the structure of metakaolin-based GPC was unconsolidated and showed poorer acid resistance than the fly ash-based ones when they possessed the same mix proportions. On the contrary, the high-calcium fly ash-based GPC activated by a high concentration of alkali showed good acid resistance.

#### 3.1.2. Neutralization Depth

**Figure 3:**
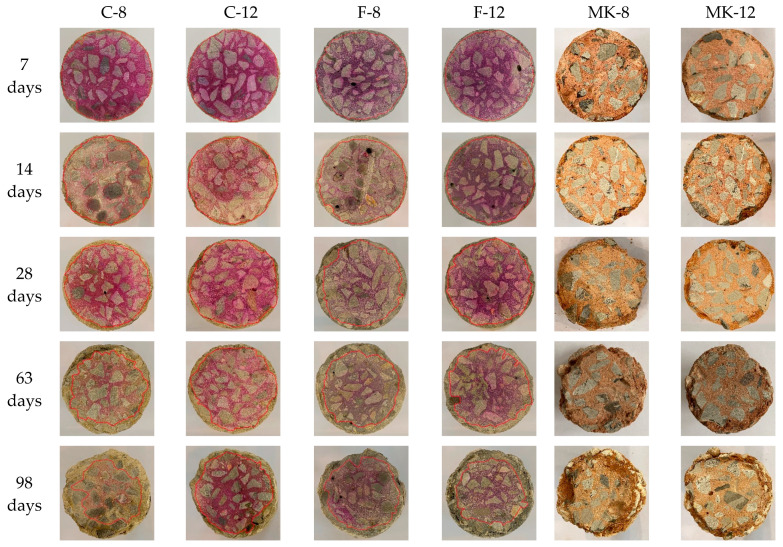
Neutralization depths of GPC.

The GPC specimen is alkaline and its section turns fuchsia when confronted with the phenolphthalein solution, but it will not display purple where the acid solution penetrates [29,30]. Figure 3 presents the variation of cross-section area of GPC specimens subjected to sulfuric acid after the immersion periods of 7 days, 14 days, 28 days, 63 days, and 98 days. When the phenolphthalein solution was sprayed on the cut surface of specimens, the portion of specimens in which there is residual alkalinity was revealed by a fuchsia color, as shown in Figure 3. It can be seen that the neutralization depths of C-8, C-12, F-8, and F-12 specimens were enlarged as their exposure time in acid solution increased. On the contrary, the specimens of MK-8 and MK-12 do not undergo the chromogenic reaction, indicating that MK specimens in this study were not resistant to sulfuric acid penetration.

**Figure 4:**
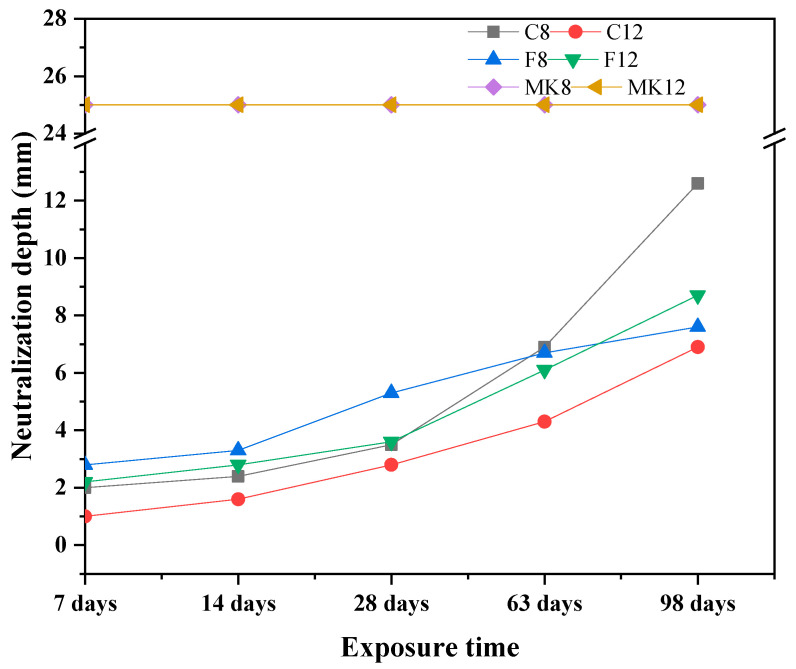
Neutralization depth of different GPCs exposed to sulfuric acid solution.

The neutralization depth variation of all GPC specimens with the increase of exposure time is displayed in Figure 4. The specimens marked with MK-8 and MK-12 were penetrated entirely by sulfuric acid at the immersion time of 7 days. This phenomenon, however, did not appear in the fly ash-based GPC specimens. This indicates that metakaolin-based GPC has a poor resistance to the acid penetration, which is consistent with the results in Figure 2. For fly ash-based GPC specimens, their neutralization depths increased with the growth of exposure time. During the first 28 days of immersion, the neutralization depths of specimens prepared with low-calcium fly ash which, is marked as F, were higher than others. After 28 days, neutralization depth of the high-calcium fly ash-based GPC specimen (C-8) dramatically increased. However, the specimen C-12 showed a slow growth depth for the neutralization depth. The neutralization depth of C-12 specimen exposed in acid solution for 98 days was only 6.9 mm, which is the minimum value. Therefore, in the light of the results of neutralization depth, the high-calcium fly ash-based GPC showed the best sulfuric acid corrosion resistance, which suggests that the increase in the Ca-content of GPC results in a higher susceptibility to the attack of sulfuric acid [31,32].

#### 3.1.3. Mass Loss

**Figure 5:**
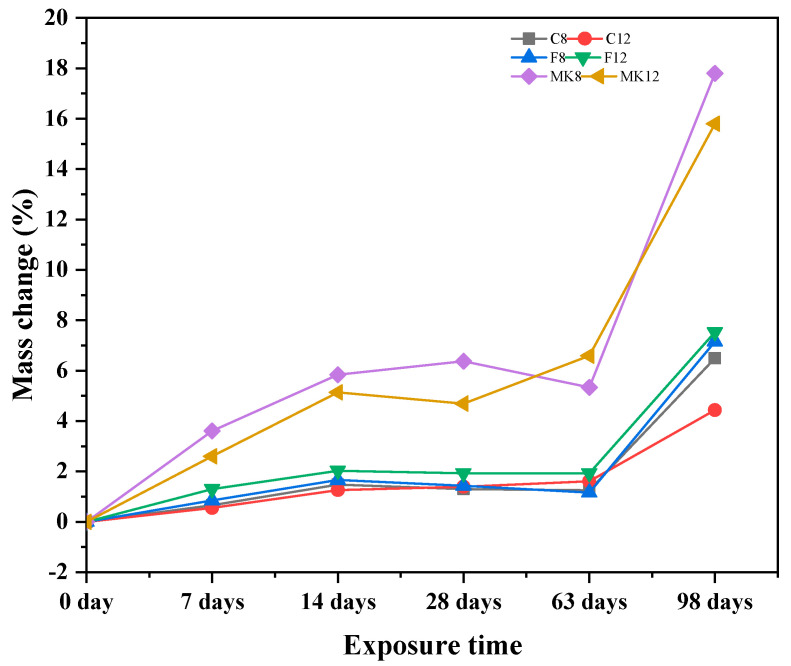
Mass loss of different GPCs exposed to sulfuric acid solution.

When the structural concrete is exposed in the acid erosion medium for a long time, its mortar will peel off from the matrix and the aggregates will be bared, leading to the mass loss of concrete and thus causing structural damage. Figure 5 shows the mass loss rate of all GPC when exposed to the sulfuric acid solution for 7, 14, 28, 63, and 98 days. As we can see from the figure, the mass loss rates of all GPC specimens sharply increased when the immersion time was up to 63 days. Before 63 days, the mass of fly ash-based GPC specimens decreased within 2%. While, the mass of metakaolin-based GPC specimens decreased more than 5%, which amounts to more than twice as much as fly ash-based GPC ones. Under the conditions of this experiment, the phenomenon illustrates that the acid resistance of the metakaolin-based GPC was far less than fly ash-based GPC. After 63 days, the mass of all GPC specimens showed a dramatic decrease when the exposure time increased. Additionally, the mass loss rate of metakaolin-based GPC reached more than 15% when the exposure time was 98 days. This result corresponds with the phenomenon in Figure 2. For the fly ash-based GPC, the mass loss rate of specimens kept within 8% at the exposure time of 98 days. However, the mass loss rate of C-12 specimen exposed in acid solution for 98 days was only 4.44%, which is the minimum value. Thus, according to the results of mass loss rate, the GPC specimen marked with C-12 showed the best sulfuric acid corrosion resistance.

#### 3.1.4. Compressive Strength

**Figure 6:**
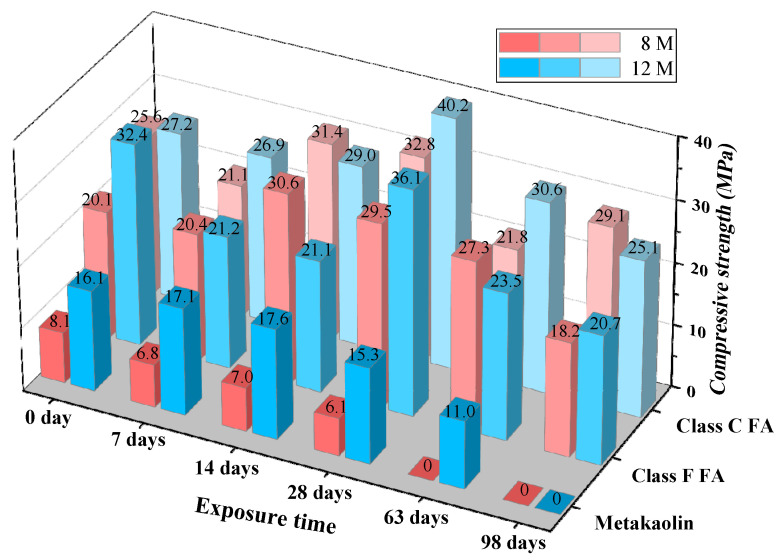
Compressive strength of GPC exposed to sulfuric acid solution.

Figure 6 reveals the evolution of compressive strength for GPC before and after exposure to the sulfuric acid solution. Initially, the compressive strengths of GPC alkali-activated by high concentration of alkaline liquor mixture (Na_2_SiO_3_ and 12 M of NaOH) were greater than that of low concentration (Na_2_SiO_3_ and 8 M of NaOH). It is attributed to the fact that the formation of geopolymeric gel depends on the concentration of alkali OH ion, but low NaOH concentration weakens the chemical reaction and reduces the compressive strength of GPC [33,34]. In addition, the compressive strength of metakaolin-based GPC specimens is too low to meet the practical engineering application, which is owing to the alkaline solution failing to promote the geopolymerization reaction thoroughly [35,36].

When the GPC specimens were exposed to sulfuric acid solution, all their compressive strength showed a tendency of rising up before coming down, except for MK-8. This is owing to that the existence of calcium that initiates the hydration mechanism, which results in the formation of calcium-based hydrated products C-S-H [25]. The co-existence of C-S-H with geopolymeric products N-A-S-H and C-A-S-H increases the compressive strength, which has been reported in previous studies [37,38,39]. The reduction in the compressive strength of GPC specimens is due to the destruction of oxy-aluminum bridge (-Al-Si-O) of geopolymeric gel when GPC suffers from sulfuric acid attack [40]. Additionally, the oxy-aluminum bridge (-Al-Si-O) is the main factor in the concrete matrix, which is responsible for strengthening the gel and increasing the bond between matrix compositions [41,42,43]. After the exposure time of 98 day, the metakaolin-based GPC specimens failed. However, the fly ash-based GPC specimens still retained partial compressive strength. In the light of the results of compressive strength, it can be found that the compressive strength of specimens made up of high-calcium fly ash and activated by high concentrations of alkaline liquor undergoing the corrosion of sulfuric acid is higher than others. This is attributed to the fact that a higher content of calcium promotes the generation of gypsum, which tends to block the pores of specimens, thus inhibiting further corrosion [44].

### 3.2. Microstructural Properties

#### 3.2.1. SEM

**Figure 7:**
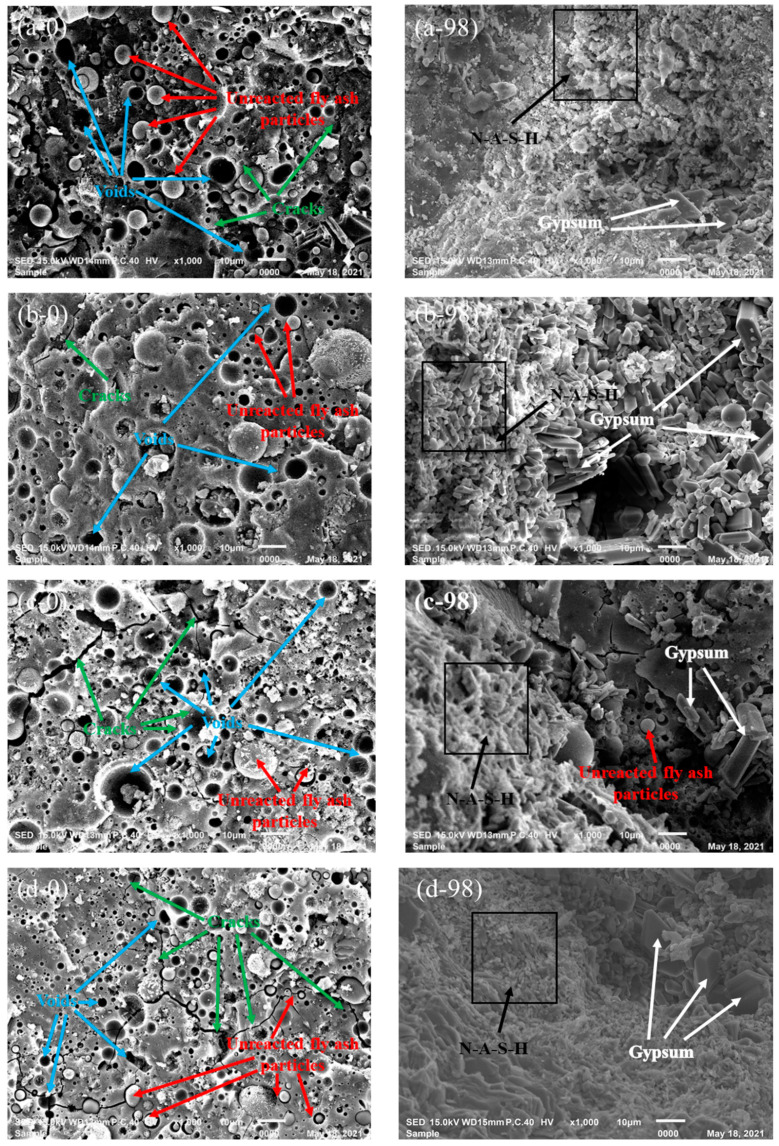

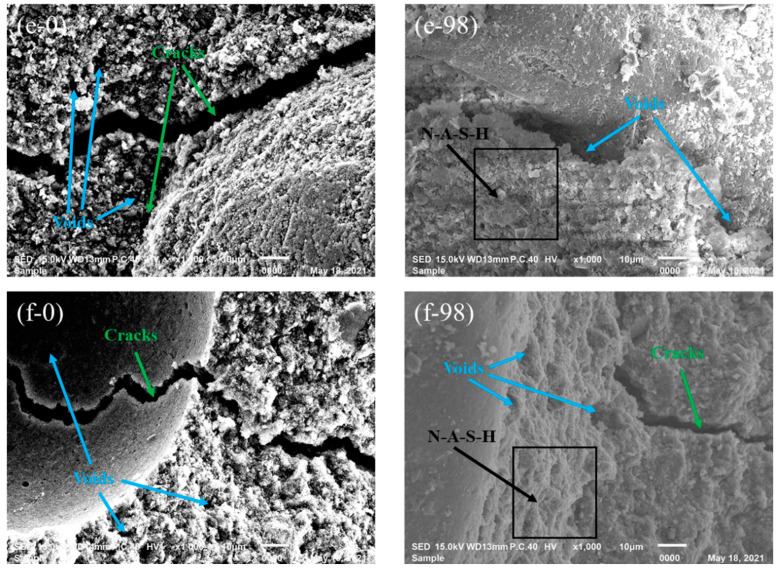
SEM images of GPC exposed to sulfuric acid solution for 0 (**left**) and 98 (**right**) days: (**a**) C-8; (**b**) C-12; (**c**) F-8; (**d**) F-12; (**e**) MK-8; (**f**) MK-12.

Figure 7 presents the SEM images of different GPC specimens exposed to the sulfuric acid solution for 0 day and 98 days. According to the results obtained from the visual appearance, neutralization depth, mass loss, and compressive strength, the maximum deterioration of all specimens was observed at 98 days of immersion. Hence, the specimens exposed to sulfuric acid solution for 98 days were observed as micrographs, and compared with the similar type of unexposed GPC. As seen from Figure 7a–d, partial unreacted fly ash particles as well as some voids and microcracks were observed in the specimens of C-8, C-12, F-8, and F-12, but the microstructures of all specimens were compact. However, the cracks of metakaolin-based GPC specimens were much larger than fly ash-based GPC ones, the microstructures were unconsolidated, and the bond between aggregate and mortar was poor (Figure 7e,f). Among the six SEM images, the fewest unreacted fly ash particles, voids, and cracks were observed in the specimens of C-12 and F-12, contributing to their higher compressive strength relative to others. This indicates that the high-concentration alkaline solution has a better activation effect in the fly-ash based geopolymerization.

Similarly, it can be seen that the specimens exposed to the sulfuric acid solution deteriorated significantly, as shown Figure 7. For the specimens exposed to sulfuric acid solution, the microstructures were looser than unexposed specimens. In general, the corrosion mechanism of OPC concrete is that sulfuric acid reacts with Ca(OH)_2_ in concrete to form gypsum, promoting the production of ettringite under certain conditions. When ettringite accumulates to a certain amount, it will expand and destroy concrete [45]. Bakharev [20] reported that the acid corrosion degradation of geopolymer materials was due to the formation of zeolites and depolymerization of geo-polymeric products. However, when comparing the morphologies of GPC specimens attacked by sulfuric acid, gypsum is found to be the most important corrosion product, except for the matrix of metakaolin. Because calcium content of high-calcium fly ash is more than low-calcium fly ash, the product of gypsum increases correspondingly. The gypsum crystals block the pores of specimens [46] and compacts the microstructure of GPC, inhibiting further corrosion of sulfuric acid [44]. The gypsum crystals in high-calcium fly ash are relatively uniform and compact, and the stress generated uniformly acts on the surrounding pore walls. On the contrary, the gypsum crystals generated by low-calcium fly ash are bulky and unevenly dispersed, which makes it easy to produce stress concentration and cause structural damage. Therefore, the high-calcium fly ash-based GPC has a better acid resistance than low-calcium fly ash-based GPC. Additionally, the acid resistance of GPC activated by 12 M NaOH is superior to the concentration of 8 M.

#### 3.2.2. X-ray Diffraction

**Figure 8:**
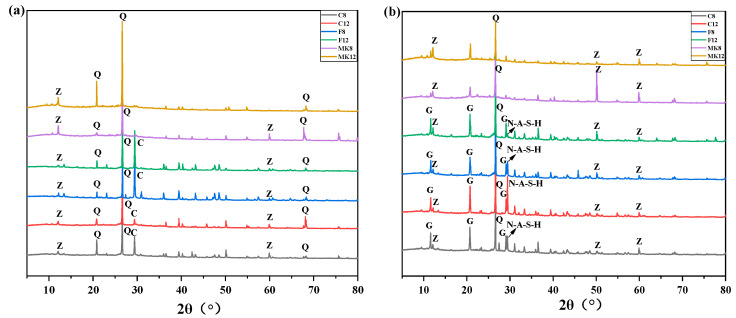
XRD patterns of GPC exposed to sulfuric acid solution for: (**a**) 0 day; (**b**) 98 days. Q = quartz, Z = gismondite, C = calcite, G = gypsum.

XRD patterns of six kinds of GPC before immersion are revealed in Figure 8a. The characteristic diffraction peaks at approximately 2θ = 20.9°, 26.6°, and 68.1° correspond to the crystal planes of quartz [47]. However, quartz was in an inert phase and generally did not react with acid solution [48]. The diffraction peak belonging to calcite was identified at 2θ = 29.5°, which was formed due to the carbonation reaction in calcium-rich binding binders [49]. Additionally, the phase of gismondite [20] was also found in the XRD spectra. By comparing the XRD patterns of unexposed GPC, the calcite [50] only existed in the matrix of high-calcium fly ash and low-calcium fly ash. It can be attributed to a certain amount of calcium oxide that existed in fly ash.

Figure 8b shows the XRD spectra of GPC exposed to the sulfuric acid solution for 98 days. The phases such as quartz, gismondite, N-A-S-H, and gypsum were identified in the XRD patterns, indicating the specimens underwent significant changes by immersion in sulfuric acid solution for 98 days. It can be seen that the peaks for calcite phases disappeared, and the new crystal phase of gypsum was generated. This can be attributed to the calcium carbonate reacting with sulfuric acid to form gypsum [51]. The diffraction peak belonging to gypsum was identified at 2θ = 11.5° and 29.2°, which has been reported in previous works [49,52]. The diffraction peak ranging from 20° to 31° was corresponding to the amorphous geopolymer phase [48], representing N-A-S-H gels. The strength of amorphous N-A-S-H gels was stronger than gismondite. Due to the intensity of peak for N-A-S-H gels appearing in metakaolin-based GPC being lower than that in fly ash-based GPC, and the metakaolin-based GPC with the higher gismondite peak, it is suggested that more gismondite than N-A-S-H gel was generated in the geopolymerization of metakaolin, contributing to the low compressive strength of metakaolin-based GPC [52,53]. In addition, the intensity of gypsum in high-calcium fly ash-based GPC was obviously higher than low-calcium fly ash based GPC, indicating the system of high calcium fly ash generated more gypsum, which is consistent with the former macro phenomenon. Besides, the peak for gypsum of GPC activated by 12 M NaOH was higher than the concentration of 8 M, which is in accordance with the results in Figure 7.

#### 3.2.3. Fourier Transform Infrared Spectroscopy

**Figure 9:**
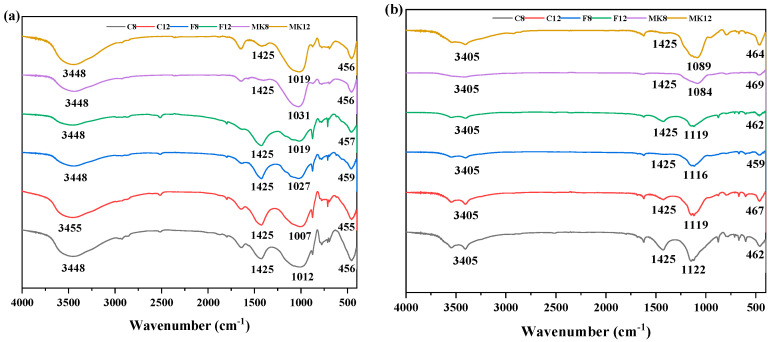
FTIR spectra of GPC exposed to sulfuric acid solution for: (**a**) 0 d; (**b**) 98 d.

Figure 9 reveals the FT-IR spectra of different GPC specimens immersed in sulfuric acid solution for 0 day and 98 days. Before immersion, the high characteristic absorption peaks at about 3448 cm^−1^ were correlated to the asymmetric stretching vibration of O–H groups in bound water [54]. It can be attributed to the fact that free water took part in the hydration during the process of geopolymerization and then turned into bound water [55]. The bands detected at 1425 cm^−1^ are attributed to tensile vibration of O–C–O bond due to the carbonation reaction [56]. This is because the geopolymer produced by NaOH activator readily absorbed CO_2_ from the atmosphere to form carbonates [55]. The geopolymerization of metakaolin -ased GPC was incomplete, resulting in an excessive amount of alkali, which is more favorable for the production of carbonates. The strongest vibration at 1007–1031 cm^−1^ is attributed to the asymmetric stretching and bending vibration of the Si–O–Si and the Al–O–Si bonds [57]. After the exposure of sulfuric acid solution, the FT-IR spectra of GPC exhibited significant changes compared with the unexposed specimens. The water component at 3448 cm^−1^ changed to 3405 cm^−1^, which was caused by the presence of gypsum [21]. After sulfuric acid attack, the vibration band at 1007–1031 cm^−1^ shifted to higher wavenumbers (1084–1122 cm^−1^). This may be attributed to the dealumination occurring in the formed gel when exposed to the acid environment [58], contributing to the decrease of intensity. Moreover, with the increase of exposure time, the vibrational bands narrowed and the area shrunk as well. However, the peak of high-calcium fly ash was still supreme. Therefore, high-calcium fly ash-based GPC suffering the corrosion of sulfuric acid solution can retain the good crystallinity of N-A-S-H gel and high residual strength.

## 4. Conclusions

Based on the results of our research, we clarified the effects of different binding materials and alkali activators of GPC on the sulfuric acid corrosion resistance. The main conclusions can be summarized as follows:

(1) Among the six kinds of GPC, the compressive strength of GPC activated by 12 M NaOH was higher than the GPC activated by 8 M NaOH, which is owing to the formation of gel depended on the concentration of alkali OH ion. The 8 M concentration of NaOH was so low that it weakened the chemical reaction and reduced the compressive strength.
(2) According to the testing results of GPC unexposed to sulfuric acid, for metakaolin-based GPC, no matter the concentration of NaOH, 8 M or 12 M, the alkali-activator will not stimulate the geopolymerization of GPC completely.
(3) Slight deterioration was observed for specimen C-12 exposed to sulfuric acid solution in terms of visual appearance, neutralization depth, and mass loss. This can be attributed to the fact that gypsum crystals block the pores of specimen and improve the microstructure of GPC, inhibiting further corrosion of sulfuric acid.
(4) From a microscopic perspective, the compressive strength of fly ash-based GPC was superior to that of metakaolin-based GPC, which can be explained from the XRD analysis, i.e., more gismondite than N-A-S-H gel was generated in the geopolymerization of the metakaolin-based geopolymer. In light of the spectra of FT-IR, high-calcium fly ash-based GPC suffering the corrosion of sulfuric acid solution can still keep the obvious peak intensity, illustrating its good crystallinity of N-A-S-H gel and high residual strength. In general, the high-calcium fly ash is more suitable to act as a binding material to prepare GPC for practical engineering.

## Figures and Tables

**Table 1:** Chemical compositions of binding materials (wt.%).

Chemical Compositions	SiO_2_	Al_2_O_3_	Fe_2_O_3_	SO_3_	TiO_2_	CaO	K_2_O	MgO	Na_2_O	LOI ^a^
Class F fly ash	44.94	32.15	5.14	2.07	1.49	9.90	1.13	1.04	0.81	1.33
Class C fly ash	44.18	26.92	9.34	1.53	1.34	11.02	1.39	1.88	1.29	1.11
Metakaolin	48.88	43.39	3.77	0.04	2.45	0.98	0.14	-	-	0.35

^a^ LOI: Loss on ignition.

**Table 2:** Proportions of mixtures (kg/m^3^).

Mixes	Binding Materials			NA	Sand	NaOH		Na_2_SiO_3_	Free Water
	Class F	Class C	Metakaolin			8 M	12 M		
F-8	377	-	-	1150	500	108	-	162	-
F-12	377	-	-	1150	500	-	108	162	-
C-8		450		1150	500	108	-	162	-
C-12		450		1150	500	-	108	162	-
MK-8	-	-	399	1150	500	108		162	60
MK-12	-	-	399	1150	500		108	162	60

## Data Availability

Not applicable.
